# Silver Nanoparticles Prepared Using *Encephalartos laurentianus* De Wild Leaf Extract Have Inhibitory Activity against *Candida albicans* Clinical Isolates

**DOI:** 10.3390/jof8101005

**Published:** 2022-09-25

**Authors:** Fatemah A. Alherz, Walaa A. Negm, Engy Elekhnawy, Thanaa A. El-Masry, Eman M. Haggag, Moneerah J. Alqahtani, Ismail A. Hussein

**Affiliations:** 1 Department of Pharmaceutical Science, College of Pharmacy, Princess Nourah bint Abdulrahman University, P.O. Box 84428, Riyadh 11671, Saudi Arabia; 2 Department of Pharmacognosy, Faculty of Pharmacy, Tanta University, Tanta 31527, Egypt; 3 Department of Pharmaceutical Microbiology, Faculty of Pharmacy, Tanta University, Tanta 31527, Egypt; 4 Department of Pharmacology and Toxicology, Faculty of Pharmacy, Tanta University, Tanta 31527, Egypt; 5 Department of Medical Microbiology and Immunology, Faculty of Medicine (Kasr Al Aini hospitals), Cairo University, Giza 12622, Egypt; 6 Department of Pharmacognosy, College of Pharmacy, King Saud University, P.O. Box 2457, Riyadh 11451, Saudi Arabia; 7 Department of Pharmacognosy and Medicinal Plants, Faculty of Pharmacy (Boys), Al-Azhar University, Cairo 11884, Egypt

**Keywords:** antifungal activity, LC-MS/MS, qRT-PCR, SEM, TEM, TNF-α

## Abstract

*Candida albicans* is a major human opportunistic pathogen causing infections, which range from cutaneous to invasive systemic infections. Herein, the antifungal and anti-biofilm potential of silver nanoparticles (AgNPs) green synthesized in the presence of *Encephalartos laurentianus* leaf extract (ELLE) were investigated. The bioactive chemicals of ELLE, including phenolics, flavonoids, and glycosides were identified and quantified for the first time. AgNPs showed minimum inhibitory concentration (MIC) values against *C. albicans* clinical isolates ranging from 8 to 256 µg/mL. In addition, AgNPs significantly decreased biofilm formation. The impact of AgNPs on the expression of the genes encoding biofilm formation was assessed using qRT-PCR. AgNPs had a beneficial role in the macroscopic wound healing, and they resulted in complete epithelization without any granulation tissue or inflammation. Treatment with AgNPs resulted in negative immunostaining of tumor necrosis factor-α. The levels of the inflammation markers, interleukin-6 and interleukin-1β, significantly decreased (*p* < 0.05) in the AgNPs-treated group. There was also a pronounced increase in the gene expression of fibronectin and platelet-derived growth factor in the wound tissues. Thus, AgNPs synthesized using ELLE may be a promising antifungal and wound healing agent.

## 1. Introduction

Nanotechnology has yielded various beneficial applications in biotechnology and microbiology. This is due to its ability to develop, design, and manipulate nanostructures with a high ratio of surface area to volume [1].

Nanoparticles have different sizes, morphologies, and chemical characteristics that can be used in their classification. Metal nanoparticles are inorganic nanoparticles, such as silver nanoparticles (AgNPs), with distinctive characteristics [2]. Their sizes range from 1 to 100 nm with high porosity and surface charge. AgNPs have received special attention owing due to their high antimicrobial activity, resulting in numerous applications designed to control the spread of pathogenic microorganisms [3]. 

There are many methods for AgNPs synthesis including physical synthesis using laser ablation or chemical synthesis involving chemical reduction with organic and inorganic reducing agents. Most of these techniques have limitations including the high cost and the use of toxic or hazardous chemicals. To overcome such drawbacks, extensive research has been done to find alternative methods that enable eco-friendly, cheap, facile, and nontoxic synthesis of AgNPs [4].

Recently, green synthesis has used eco-friendly non-toxic reagents from plants and microorganisms to reduce silver ions. The use of natural extracts may be more advantageous than microorganisms for the synthesis of nanoparticles because complex and specific processes like culture maintenance and isolation are not needed [5].

Different plant parts can be utilized for the biosynthesis of nanoparticles, such as leaves, flowers, roots, and fruits. *Encephalartos* are among the plants that are not well studied. They belong to the Cycadales, the second-largest extant genus. *Encephalartos laurentianus* is the largest cycad and has recently been found to possess cytotoxic, antioxidant, and antibacterial characteristics [6]. We used the leaf extract from this plant to synthesize AgNPs as *E. laurentianus* contains a variety of active compounds, including flavonoids, flavonoid glycosides, and phenolic and organic acids [7]. 

Various reports of the biological activities of AgNPs led us to assess their activity against *Candida albicans* clinical isolates. *C. albicans* is among the most commonly encountered fungal pathogens and causes high morbidity and mortality, particularly among immunocompromised and critical care patients [8]. While *C. albicans* is a commensal of the oral cavity, gastrointestinal tract, and vagina, it is an opportunist able to produce severe systemic infections. In addition, *C. albicans* can infect wounds, which can result in invasive candidiasis [9].

There is an urgent need to identify new antifungal agents against *C. albicans* as the current antifungal drugs suffer from many disadvantages including drug resistance, narrow spectrum, and significant side effects [10]. This report describes the synthesis of AgNPs using ELLE, as a capping agent, and investigates their in vitro and in vivo antifungal activities against *C. albicans*. In addition, LC-MS/MS was used to identify the potential bioactive components of ELLE, involved in the synthesis of AgNPs. 

## 2. Materials and Methods

### 2.1. Chemicals and Preparation of Plant Extract

*Encephalartos laurentinus* De Wild. leaves (Family Zamiaceae) were obtained from Al-Abid Farms on 13 January 2017. *E. laurentianus* was identified by Dr. Esraa Ammar, Tanta University. The leaves were dried at room temperature for two weeks and then ground. The powder (400 g) was extracted using the cold maceration method with methanol (4 L × four times till complete exhaustion, two-day intervals) and then concentrated under reduced pressure to yield 39.6 g of ELLE [7]. 

Betadine™ ointment was purchased from Mundi pharma, Cairo, Egypt. Culture media (including Sabouraud dextrose agar (SDA), brain heart infusion (BHI), and Sabouraud dextrose broth (SDB) were purchased from Oxoid, Hampshire, UK. All other chemicals used in the study were obtained from Merck, New Jersey, USA.

### 2.2. Animals

Thirty white male Wistar albino rats (180–210 g, eight weeks old) were obtained from the Faculty of Veterinary Medicine (Cairo University). They were housed in a pathogen-free environment at 25 ± 2 °C with a 12-h light/dark cycle. They were provided with a standard pellet diet (*ad libitum* feeding) and filtered water. The rats were acclimatized for one week prior to the in vivo experiments. The authorized standards of the Faculty of Pharmacy Research Ethical Committee (Tanta University) for the use of the laboratory animals, with code number TP/RE/3-22-P-006, were used.

### 2.3. Liquid Chromatography Analysis

Liquid chromatography-electrospray ionization–tandem mass spectrometry (LC-ESI-MS/MS) of the samples used an Exion LC AC system for separation and SCIEX Triple Quad 5500+ MS/MS system equipped with electrospray ionization (ESI) for detection. The separation was performed using ZORBAX SB-C18 Column (4.6 × 100 mm, 1.8 µm). The mobile phases were mainly from two eluents, A: 0.1% formic acid in water and B: acetonitrile (L.C. grade). The mobile phase was programmed as follows: 2% B from 0–1 min, 2–60% B from 1–21 min, 60% B from 21–25 min, and 2% B from 25.01–28 min. The flow rate was 0.8 mL/min, and the injected volume was 3 µL. For multiple reaction monitoring (MRM) analysis of the selected polyphenols, positive and negative ionization modes were applied in the same run with the following parameters: curtain gas: 25 psi; ion spray voltage: 4500 and 4500 V for positive and negative modes, respectively; source temperature: 400 °C; ion source gas 1 and 2 were 55 psi with a declustering potential (DP): 50; collision energy: 25 eV; collision energy spread: 10 times [11].

### 2.4. Green Synthesis of AgNPs

Aqueous silver nitrate solution (AgNO_3_, 1 mM) was stored in a cold, dark place. Then, ELLE (10 mL) was added to AgNO_3_ solution (90 mL), and the mixture was incubated overnight in a dark environment to reduce Ag^+^ ions. The formation of a yellowish-brown tint suggested the production of AgNPs. The resultant solution was assessed using a transmission electron microscope (TEM) and ultraviolet-visible spectroscopy (UV-Vis). AgNPs were purified via centrifugation at 11.000× *g* for 30 min followed by washing three times with distilled water and filtration with filter paper [12]. 

### 2.5. Characterization of AgNPs

#### 2.5.1. UV-Vis Spectroscopy

The production of AgNPs was confirmed using a UV/Vis spectrophotometer (SHIMADZU, Kyoto, Japan) at a wavelength of 300–900 nm.

#### 2.5.2. Fourier-Transform Infrared (FTIR)

FTIR analysis was used to determine the functional groups of the plant metabolites involved in the reduction process of silver. The attenuated total reflectance mode of a Jasco FTIR4100 spectrometer (SHIMADZU, Kyoto, Japan) in 4000–400 cm^−1^ was used [13].

#### 2.5.3. High-Resolution (HR-TEM) and Scanning Electron Microscope (SEM)

HR-TEM (JEOL JEM-2100, Tokyo, Japan) and selected area electron diffraction (SAED) were used to determine the average particle size and the morphology of ELLE-capped AgNPs. SEM (TM1000, Hitachi, Tokyo, Japan) was used to examine the morphology of AgNPs [14].

#### 2.5.4. Zeta Potentials and Dynamic Light Scattering (DLS)

DLS analysis was carried out using zeta-sizer (Malvern, UK) to determine the entire core size of the AgNPs and their charge.

#### 2.5.5. X-ray Diffraction (XRD)

XRD analysis was used to confirm the occurrence of the crystal nature of AgNPs and determine their structure and size. XRD was performed on AgNPs using Cu-Kα1 X-ray diffractometer radiation (θ = 1.5406 Å) operating at 45 kV and 30 mA with 2θ in the range of 4.01–79.99° [15].

### 2.6. Determination of Total Flavonoids and Phenolics of ELLE

Colorimetric analysis of ELLE dilution series was carried out using the aluminum chloride procedure with Rutin as a reference to assess the total flavonoid concentration [15]. The content of polyphenols was evaluated using the Folin-Ciocalteu technique with gallic acid as the reference [16].

### 2.7. Antioxidant Activity of ELLE

#### 2.7.1. ABTS Radical Scavenging Capacity

The 2,2′-azino-bis (3-ethylbenzothiazoline-6-sulfonic acid) (ABTS) assay was conducted as previously described [17]. The results are expressed as µM Trolox equivalents (TE)/mg of samples using the linear regression equation derived from the calibration curve. 

#### 2.7.2. Ferric Reducing Antioxidant Power (FRAP) Assay

FRAP assay was performed as previously reported. The results were expressed using the linear dose-response curve of Trolox [18].

### 2.8. Antifungal Potential (In Vitro)

#### 2.8.1. Fungi

Thirteen *C. albicans* clinical isolates were collected from patients admitted to different departments of Tanta University Hospitals, Egypt. The samples were obtained from the patients for laboratory diagnosis and were not collected specifically for this study. The clinical specimens (skin swabs) were first cultured on SDA plates, then incubated overnight at 37 °C. The cultures were identified as *C. albicans* using matrix-assisted laser desorption/ionization-time of flight (MALDI-TOF) (bioMerieux, Marcy-l’Étoile, France) with *Candida albicans* (MTCC 227) used as the standard isolate. 

#### 2.8.2. Antifungal Activity

The disc agar diffusion method for screening the antifungal potential of the green synthesized AgNPs was used as previously described [7,19]. In brief, sterile filter paper discs impregnated with AgNPs were added after spreading the fungal suspension on the SDA plates. Each plate included a positive control (fluconazole, 10 µg) and negative control (dimethyl sulfoxide, DMSO, 10%). The plates were then incubated at 37 °C for 24 h and inspected for the appearance of inhibition zones around the discs.

#### 2.8.3. Estimation of Minimum Inhibitory Concentrations (MICs)

The MIC values of AgNPs against *C. albicans* isolates were estimated using the broth microdilution method in a 96-well microtitration plate using a two-fold serial dilution of AgNPs [20]. A positive control (untreated fungi) and a negative control (SDB only) were included in each microtitration plate. After incubation at 37 °C for 24 h, the minimum concentration of AgNPs, which caused a complete absence of the growth of *C. albicans* cells, was recorded as MIC [21,22].

#### 2.8.4. Time Kill Assay

It was carried out as previously reported [23]. Overnight cultures of *C. albicans* isolates in BHI broth were adjusted to 10^6^ CFU/mL. Then, AgNPs (0.5× MIC, 1× MIC, 2× MIC, 4× MIC, and 8× MIC values) were added and optical density (OD) values, at 530 nm, were determined after 4, 8, 24, 28, 32, and 48 h. 

#### 2.8.5. Biofilm Formation Assay

Overnight *C. albicans* cultures in SDB were adjusted to 1 × 10^6^ CFU/mL, and 100 μL was added to the wells of the microtiter plates and incubated for 48 h at 37 °C. The wells were rinsed gently with phosphate-buffered saline (PBS) and the fungal cells that adhered to the wells were fixed using 100 μL methanol for 20 min. *C. albicans* cells were stained using 1% crystal violet for 20 min, the wells then gently washed with distilled water and allowed to dry. The bound dye was dissolved using glacial acetic acid (33%), and the OD values, at 540 nm, were measured using an ELISA reader (Sunrise Tecan, Zürich, Switzerland) [11]. The classification of *C. albicans* isolates according to their biofilm-forming ability is shown in Table S1. 

#### 2.8.6. Anti-Biofilm Activity

The anti-biofilm effect of AgNPs (at 0.5 MIC values) against *C. albicans* isolates moderately and strongly biofilm-forming was measured using a crystal violet assay as previously described [7]. The number of colony-forming units (CFU/mL) was used to assess the viability of the fungal cells forming biofilms. The cells forming biofilms were gently rinsed with PBS and then scraped off the wells using toothpicks after adding 200 μL PBS. The fungal suspensions were serially diluted and plated on SDA plates supplemented with chloramphenicol, and the number of CFU/mL was determined after overnight incubation at 37 °C. 

#### 2.8.7. SEM

Glass coverslips submerged with *C. albicans* isolates (before and after treatment with AgNPs) were overnight incubated at 37 °C to permit the fungal cells to form biofilms. The coverslips were gently rinsed with PBS, flooded with 2.5% glutaraldehyde solution, left overnight at 4 °C, and dehydrated using a series of ethanol concentrations (30% to 100%). They were allowed to air dry and coated with gold for examination using SEM (Hitachi, Tokyo, Japan).

#### 2.8.8. Quantitative Real-Time PCR (qRT-PCR)

The expression of the biofilm encoding genes (*BCR*1, *PLB*2, *ALS*1, and *SAP*5) were evaluated, using qRT-PCR, after treatment with AgNPs [12]. After extracting the total RNA using an RNeasy mini kit (Qiagen, Hilden, Germany), cDNA was synthesized using the SensiFAST™ cDNA kit (Bioline, London, UK). The qRT-PCR was carried out using SensiFAST™ SYBR green PCR master mix (Bioline, London, UK) as described by the manufacturer. All reverse-transcription experiments included a negative control to test for contaminating genomic DNA. The negative control contained all components except for the template RNA. The used primers are presented in Table S2 using *ACT*1 as a housekeeping gene. The expression of the tested genes (*BCR1*, *PLB2*, *ALS1*, and *SAP5*) in *C. albicans* isolates before treatment with AgNPs was considered to have a value of one. Relative gene expression was calculated using the 2^−ΔΔCT^ method [8]. Fold changes were considered to be statistically significant when there were two or more fold changes (either increased or decreased) [24]. 

### 2.9. In Vivo Antifungal Potential

#### 2.9.1. Experimental Model

Rats were randomly grouped into three equal groups. These groups were: group I (control, 0.9% normal saline), group II (Betadine™ ointment), and group III (AgNPs solution, 5 μg/mL). After being anesthetized, a limited skin area on the backs of the rats was carefully shaved. Full thickness excisional wounds, with an initial size of 1.2 ± 0.2 cm^2^, were prepared and inoculated with *C. albicans* (C3 isolate, 10^6^ CFU/mL). Betadine™ ointment, AgNPs, or 0.9% normal saline were applied topically on the wound’s surface daily for seven days.

#### 2.9.2. Macroscopic Features of the Wound

From day zero (the day of wound creation), the process of wound healing was observed for seven days. Digital images of the wounds were recorded on days zero, three, and seven. The wound areas (cm^2^) were measured using a ruler on these days to assess healing efficacy [25]. The wound healing percentage was calculated using the following equation:

$$\mathrm{Wound}\;\mathrm{healing}\;\mathrm{percentage}=\frac{\left(\mathrm{wound}\;\mathrm{area}\;\mathrm{at}\;\mathrm{day}\;\mathrm{zero}-\mathrm{wound}\;\mathrm{area}\;\mathrm{at}\;n\mathrm{th}\;\mathrm{day}\right)\times 100\;}{\mathrm{wound}\;\mathrm{area}\;\mathrm{at}\;\mathrm{day}\;\mathrm{zero}}$$

*n* represents day three or day seven.

#### 2.9.3. Fungal Burden

The fungal burden was calculated on days three and seven post wounding. Excised wound tissues were homogenized in 10 mL PBS, serially diluted (10-fold) dilutions in SDB, and plated onto SDA plates, supplemented with chloramphenicol antibiotic to prevent bacterial growth. The plates were incubated at 37 °C for 24 h for quantification of CFU/mL.

#### 2.9.4. Histopathological Examination

The entire wound, with a five-millimeter margin of the surrounding intact skin, was obtained from each rat at the end of the experiment for histological assessment [26]. The specimens were fixed with formalin solution (10%) and inserted into paraffin wax at 65 °C for block formation. The tissue blocks were sliced into thin sections, stained with hematoxylin and eosin (H&E) and Masson’s trichrome stain (for collagen staining) and inspected using a light microscope [27]. The percentage of the area of collagen fibers was assessed using image J software (National Institutes of Health, Bethesda, MD, USA).

#### 2.9.5. Immunohistochemistry

The thin sections were dewaxed, rehydrated, and stained with tumor necrosis factor-alpha (TNF-α) antibody (ABclonal Technology, Woburn, MA, USA). The strength of staining was graded as negative (−) if there were not any positive cells, mild (+) if the positive cell percentage ranged from 1% to 10%, moderate (++) if the positive cell percentage ranged from 11% to 50%, and strong (+++) if the positive cell percentage was more than 50% [10].

#### 2.9.6. ELISA

Interleukin-1β (IL-1β) and (IL-6) levels were assessed in the wound tissues (pg/mg tissues) using an ELISA kit (Abcam Co., Waltham, MA, USA). 

#### 2.9.7. Gene Expression of Fibronectin and Platelet-Derived Growth Factor (PDGF) Genes

qRT-PCR was carried out as previously described using the glyceraldehyde-phosphate dehydrogenase (GAPDH) gene as a reference gene. The relative expression of fibronectin and PDGF genes was determined (primers listed in Table S3) [25].

#### 2.9.8. Statistical Analysis

All assays were performed in triplicate, and the results are presented as mean ± standard deviation (SD). The results were regarded as statistically significant at *p* < 0.05 using the GraphPad Prism 8 (USA) via ANOVA and a post hoc test. 

## 3. Results

### 3.1. LC-ESI-MS/MS Analysis

**Figure 1:**
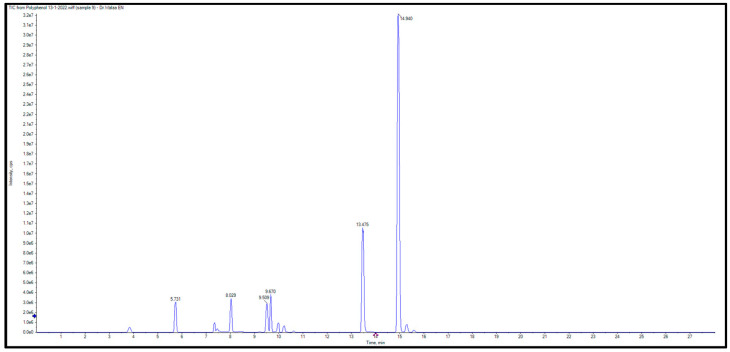
Liquid chromatography-electrospray ionization–tandem mass spectrometry (LC-ESI-MS/MS) of ELLE.

Liquid Chromatography with tandem mass spectrometry LC/MS was employed to identify and quantify phenolic chemicals in ELLE. The LC-ESI-MS/MS chromatogram for ELLE’s identified flavonoids and phenolics is shown in Figure 1. Ten phenolics were identified, which were (gallic, 3,4-dihydroxybenzoic, chlorogenic, methyl gallate, caffeic, syringic, coumaric, vanillic, ellagic, and ferulic acid). In addition, seven flavonoids were detected (rutin, luteolin, quercetin, naringenin, apigenin, kaempferol, and hesperetin). The LC-MS/MS analysis of *E. laurentianus* found in Table 1 explored the presence of naringenin at a concentration of 66,647 μg/g as the major flavonoid, followed by luteolin (486 μg/g). The main phenolic acid was 3,4-dihydroxybenzoic acid (767 μg/g), followed by chlorogenic acid (106 μg/g).

### 3.2. Characterization of the AgNPs

#### 3.2.1. UV-Vis Spectroscopy

**Figure 2:**
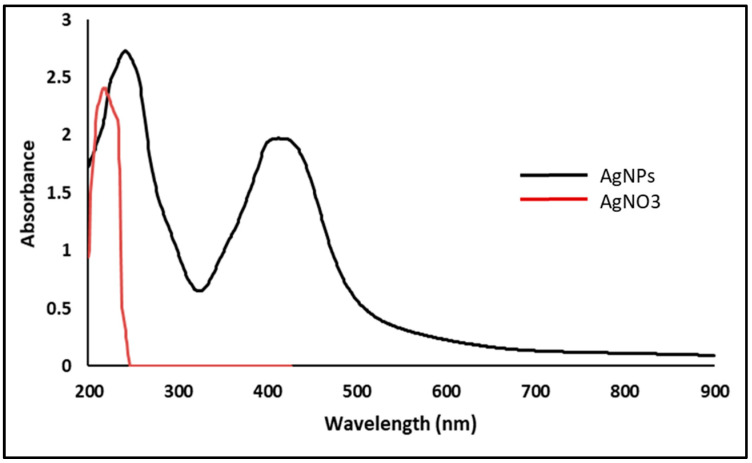
UV spectrum of AgNPs.

It was utilized to demonstrate AgNPs creation because of UV’s selectivity for the produced AgNPs. AgNPs interact strongly with specific wavelengths of light due to their distinctive optical reflectivity. For example, free electrons create a surface plasmon resonance (SPR) absorption band in AgNPs due to collective electron oscillation [28]. An UV absorbance peak in the ELLE-capped nanoparticles was detected at 432 nm (Figure 2). 

#### 3.2.2. FTIR

**Figure 3:**
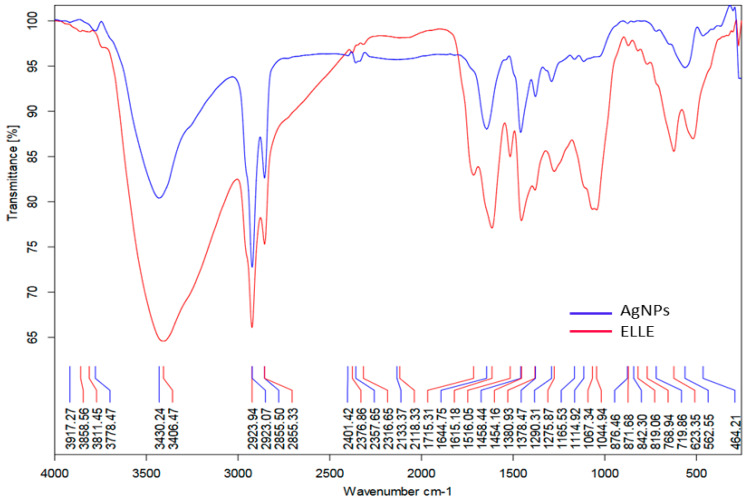
FTIR spectrum of AgNPs and ELLE.

The presence of an OH group in the phenolic compounds was confirmed via FTIR data for ELLE (Figure 3), which showed multiple bands at 3430 cm^−1^. The medium peak shows a stretch alkane group at 2923 cm^−1^ (CH). CH bending vibrations and C-C or C=O stretch in the phenolic components caused the bands in the ELLE spectra in 1713, 1624, 1516, and 1378 cm^−1^. Another intense band at 842 cm^−1^ was characteristic at the aromatic ring. Owing to the phenolic and amino groups attachment, these bands’ frequencies reduced in strength attachment via AgNPs compared with ELLE [29]. 

ELLE contains flavonoids, glycosides, phenolics, and organic acids components that might cap the AgNPs. From the phytochemical results of LC-ESI-MS/MS, we suggest that -OH (hydroxyl), -C=O (Carbonyl) of ELLE also had a role in AgNPs production.

#### 3.2.3. TEM and SEM

**Figure 4:**
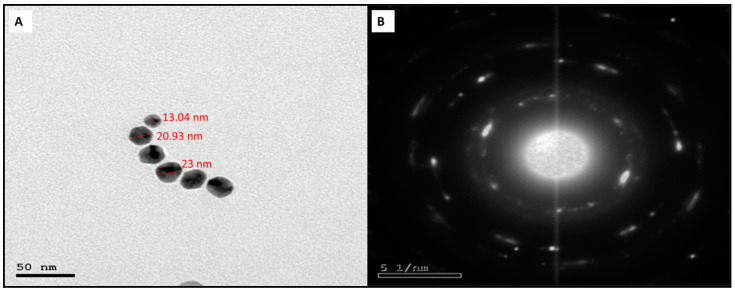
Electron micrographs of the green synthesized AgNPs capped with ELLE. (**A**) TEM and (**B**) SAED.

**Figure 5:**
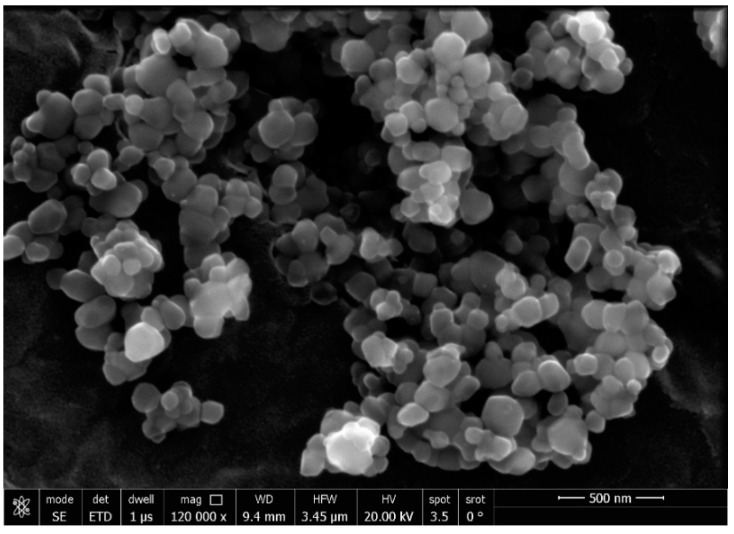
SEM of AgNPs capped with ELLE.

The production of AgNPs with a spherical shape and a particle size range of 13.04–23.0 nm, with an average size of 18 ± 5 nm, was observed employing HR-TEM on AgNPs (Figure 4). The crystallinity of the AgNPs was confirmed by the sharp ring diffraction pattern detected with SAED. SEM analysis showed the AgNPs had a spherical shape and a tendency to aggregate (Figure 5).

#### 3.2.4. Zeta Potential and DLS

**Figure 6:**
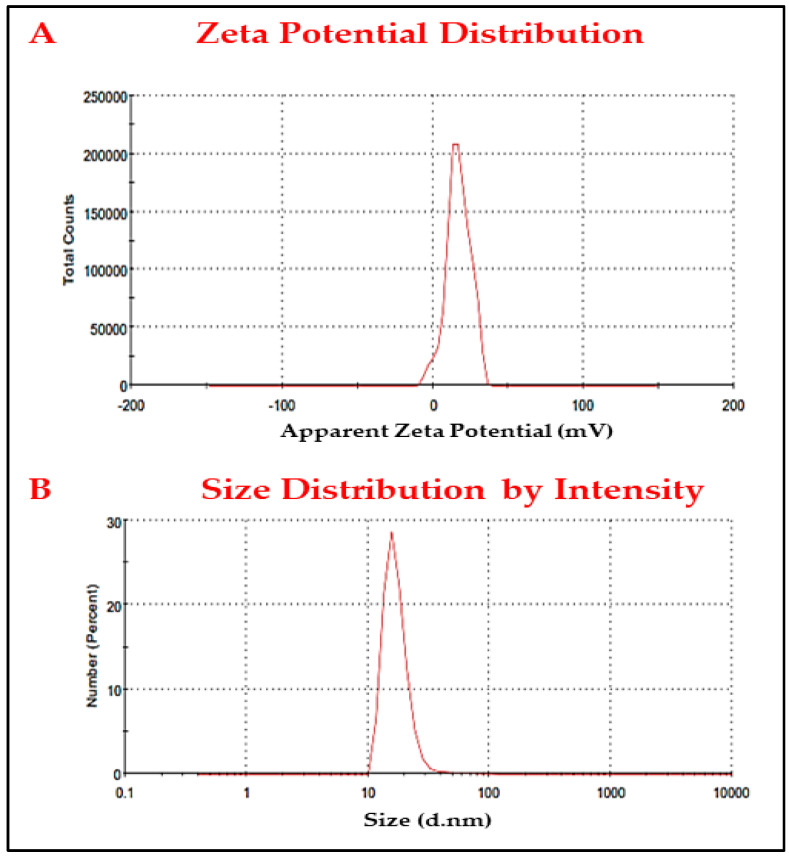
(**A**) ζ−potential analysis and (**B**) DLS of the synthesized AgNPs using ELLE.

The surface charge of the synthesized AgNPs was determined using the ζ-potential approach. AgNPs have a mean ζ-potential of −7.04 ± 0.7 mV, with the negative charge highlighting the nanoparticles’ stability (Figure 6A). The DLS technique was used to measure the AgNPs size distribution, which included the metallic shell of the nanoparticles. AgNPs had a mean size of 78.4 ± 3.8 nm and a PDI value of 0.406 (Figure 6B). 

#### 3.2.5. X-ray Diffraction

**Figure 7:**
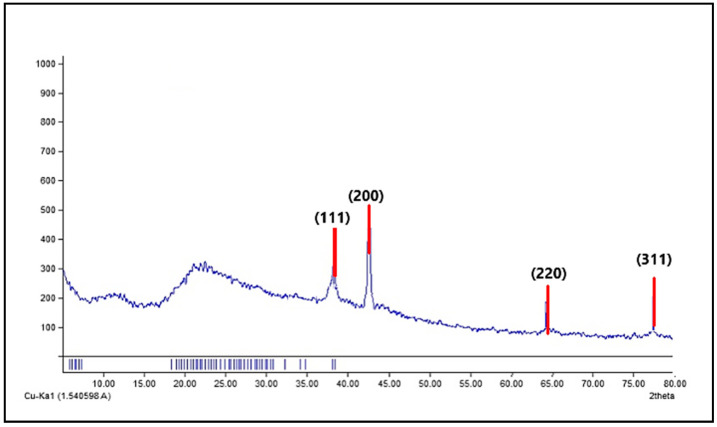
X-ray diffraction (XRD) pattern of the green synthesized AgNPs by ELLE.

The intense diffraction peaks were observed at the 2θ values of 38.44, 44.47, 64.35, and 77.94, corresponding to (111), (200), (220), and (311) planes for AgNPs, respectively (Figure 7). Remarkably, there are no other peaks, which implies that the AgNPs were pure.

### 3.3. Flavonoids and Phenolics’ Total Content

The total flavonoids content of ELLE was 17.38 ± 0.95 µg rutin equivalent/mg sample, and the total polyphenols content was 95.89 ± 2.42 µg gallic acid/mg sample. These findings indicate that ELLE has high contents of phenols and flavonoids.

### 3.4. Antioxidant Activity of ELLE

The radical scavenging and metal-reducing assays were used to explore ELLE’s antioxidant effects. According to ABTS, ELLE has an antioxidant activity of 530.73 µM Trolox equivalents (TE)/mg. The FRAP test was utilized as a metal-reduction assay, and the ELLE activity was 189.87 µM TE/mg.

### 3.5. In Vitro Antifungal Potential

AgNPs showed antifungal potential against *C. albicans* isolates with formation of inhibition zones like fluconazole. The MIC values of AgNPs against the tested *C. albicans* isolates ranged from 8 to 256 µg/mL (Table S4). 

#### 3.5.1. Time Kill Curve

**Figure 8:**
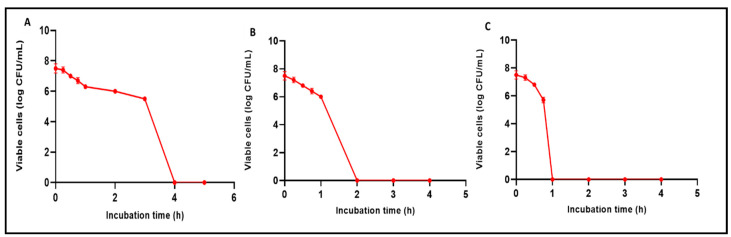
Charts representing the decrease in the number of CFU/mL of *C. albicans* isolates after incubation with AgNPs: (**A**) with concentrations of 2× MIC for four hours, (**B**) 4× MIC for two hours, and (**C**) 8× MIC for one hour.

The CFU/mL of *C. albicans* isolates decreased by at least three log units after incubation with AgNPs at concentrations of 2× MIC for four hours, 4× MIC for two hours, and 8× MIC for one hour for 69.23%, 53.85%, and 30.77% of *C. albicans* isolates, respectively. An illustrative example is presented in Figure 8. 

#### 3.5.2. Antibiofilm Activity

**Figure 9:**
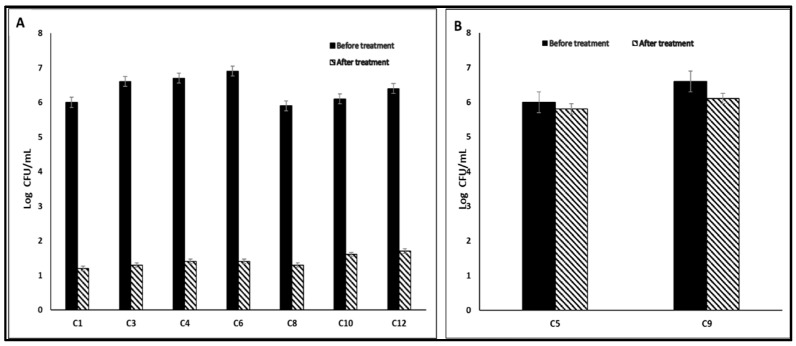
Bar chart showing (**A**) a significant decrease (*p* < 0.05) and (**B**) non-significant change (*p* > 0.05) in the number of CFU/mL after AgNPs treatment.

The impact of AgNPs on the biofilms of *C. albicans* isolates was assessed via a crystal violet test. The AgNPs reduced the percentage of *C. albicans* isolates, which form biofilm strongly and moderately (C1, C3, C4, C5, C6, C8, C9, C10, and C12), from 69.23% to 30.77% (Table 2). Furthermore, AgNPs decreased significantly (*p* < 0.05) the number of CFU/mL in 53.85% of *C. albicans* isolates (Figure 9).

#### 3.5.3. SEM

**Figure 10:**
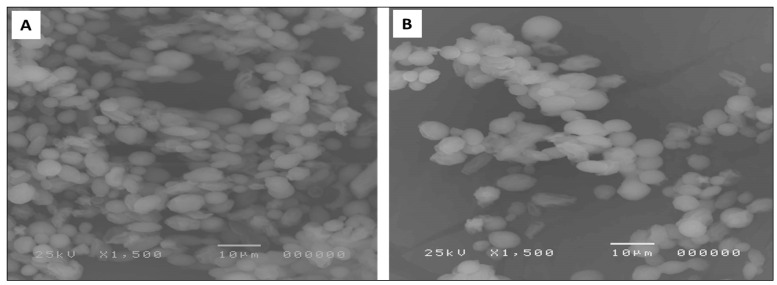
SEM micrograph showing biofilm formation by *C. albicans* (C6) isolate (**A**) before and (**B**) after AgNPs treatment.

SEM was used to study the effect of the biosynthesized nanoparticles on the biofilm morphology. A remarkable decrease in the biofilms formed by *C. albicans* isolates was found after treatment with AgNPs in five isolates (38.46% of isolates). An illustration of the visible inhibition of the biofilm formation is shown in Figure 10. 

#### 3.5.4. qRT-PCR

**Figure 11:**
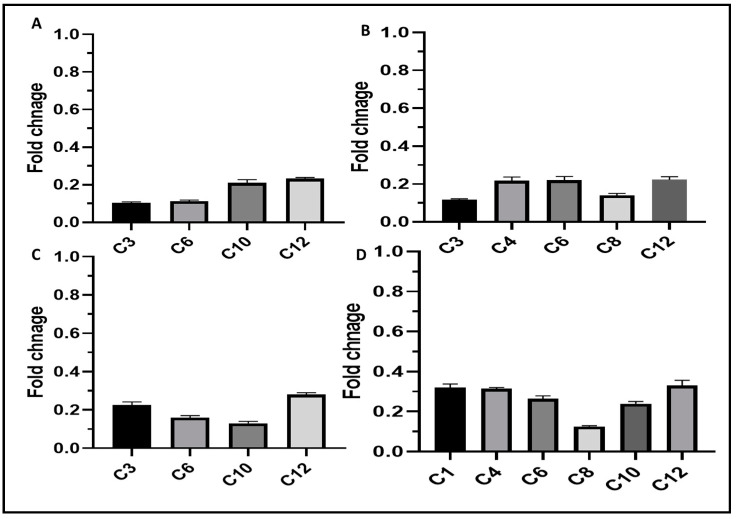
Bar charts showing the downregulation caused by AgNPs in the expression of (**A**) *BCR*1, (**B**) *PLB*2, (**C**) *ALS*1, and (**D**) *SAP*5 genes in *C. albicans* isolates.

Relative gene expression of *BCR*1, *PLB*2, *ALS*1, and *SAP*5 biofilm genes was determined via qRT-PCR in *C. albicans* isolates to assess the influence of AgNPs on biofilm formation. As shown in Figure 11, a significant downregulation of the expression of *BCR*1, *PLB*2, *ALS*1, and *SAP*5 was detected in 30.77%, 38.46%, 30.77%, and 46.15% of the isolates, respectively. 

### 3.6. In Vivo Antifungal Potential

#### 3.6.1. Macroscopic Wound Healing and Fungal Cell Viability

The Betadine™- and AgNPs-treated groups exhibited an entire and prominent wound healing process relative to the control group (Figure S1). 

**Figure 12:**
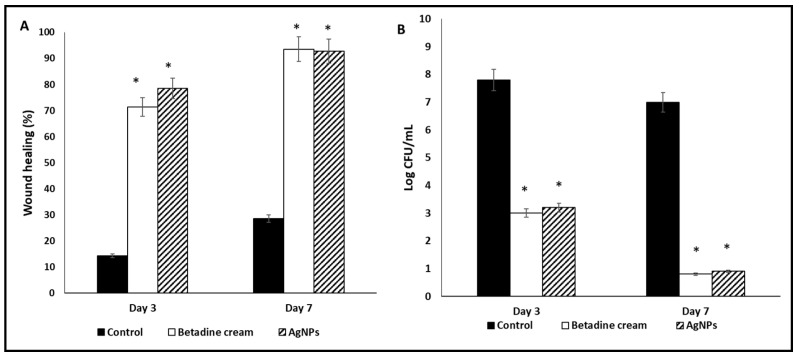
Characters of the wounds on days 3 and 7: (**A**) wound healing percentage and (**B**) log CFU/mL. The symbol (*) represents a significant change (*p* < 0.05) in comparison with the control group.

The rats of Betadine™- and AgNPs-treated groups showed remarkable wound healing on day three (percentages of wound healing were 71.43% and 78.57%, respectively) in comparison with the control group. The two groups revealed complete wound healing on day seven, with percentages of wound healing of 93.57% and 92.86%, respectively (Figure 12A). Moreover, the number of viable *C. albicans* cells (CFU/mL) were several orders of magnitude lower in the Betadine™- and AgNPs-treated groups compared to the control group (Figure 12B).

#### 3.6.2. Histopathology

**Figure 13:**
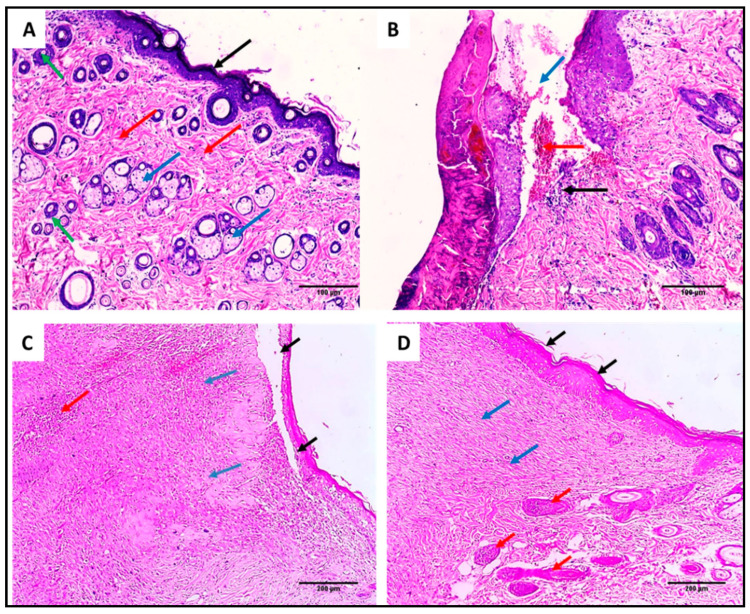
H&E-stained wound sections of (**A**) section in normal skin showed normal epidermal thickness (black arrow), with underlying dermis showing thick collagen bands (red arrows), hair follicles (green arrows), and sebaceous glands (blue arrows) (×200). (**B**) Section in the wound of the control group showed an ulcer (blue arrow) filled with hemorrhage (red arrow) and acute and chronic inflammatory cellular infiltrate (black arrow) (×200). (**C**) Section in the Betadine™-treated group showed partial epithelization (black arrow) with underlying granulation tissue (blue arrows) with little inflammatory cellular infiltrate (red arrow) (×200). (**D**) Section in the AgNPs-treated group showed complete epithelization (black arrows) with underlying focal granulation tissue formation (blue arrows) with adjacent proliferating hair follicles (red arrows) without inflammation (×200).

Normal skin sections revealed normal epidermal thickness, with the underlying dermis showing thick collagen bands, hair follicles, and sebaceous glands (Figure 13A). In contrast, the wound sections of the control group revealed the presence of ulcers filled with hemorrhage accompanied by acute and chronic inflammatory cellular infiltrate (Figure 13B). The wound section of the Betadine™ group exhibited a complete epithelization with underlying granulation tissue without any inflammatory cellular infiltrate (Figure 13C). Wound sections of the AgNPs-treated group also showed a complete epithelization with underlying dermis containing thick collagen bundles and proliferating hair follicles without any granulation tissue or inflammation (Figure 13D). 

**Figure 14:**
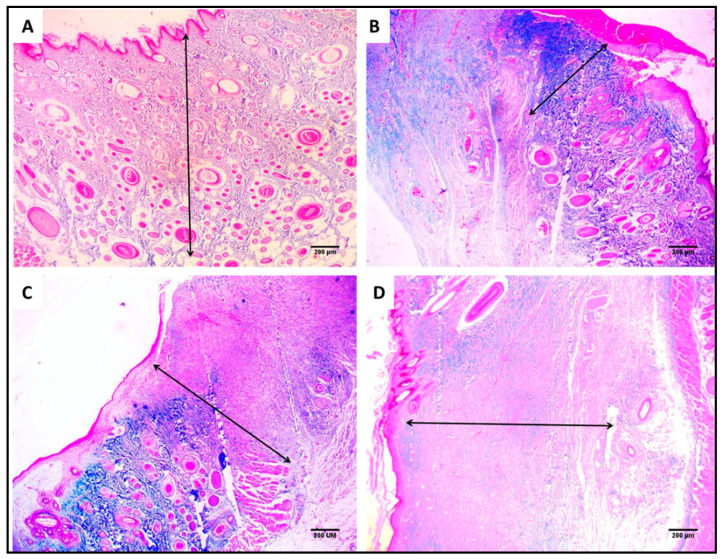
Masson’s trichrome staining of the wound showing the area of collagen fibers (%) using image J software of (**A**) section in normal skin (negative control) showing 28.2% area of collagen fiber (×40). (**B**) Section in the wound of the control group showing 8.2% area of collagen fiber (×40). (**C**) Section in the Betadine™-treated group showing 14.7% area of collagen fiber (×40). (**D**) Section in the AgNPs-treated group showing 22.4% area of collagen fiber (×40).

Light microscopy of Masson’s trichrome-stained wound sections was used to inspect the collagen fibers (Figure 14). The percentages of the collagen fibers in the Betadine™- and AgNPs-treated groups were significantly higher than the control group.

#### 3.6.3. Immunohistochemistry

**Figure 15:**
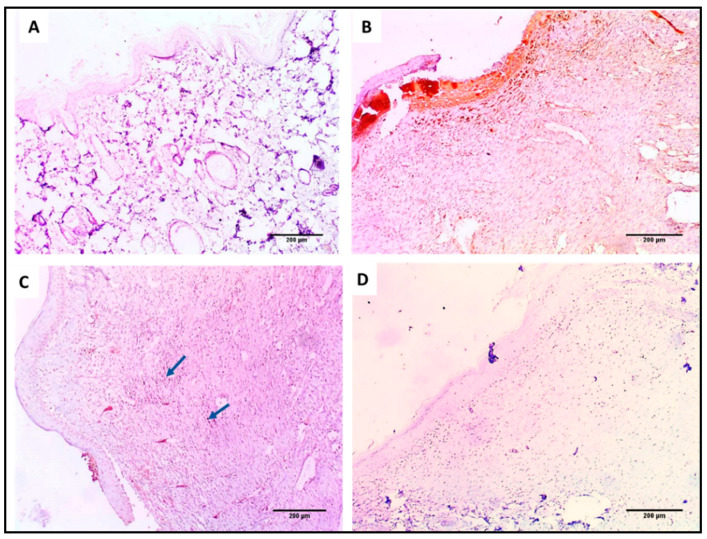
TNF-α immunohistochemical staining of (**A**) section in normal skin (negative control) showing negative TNF-α immunostaining (−) as no wound healing area (×100). (**B**) Section in the wound of the control group showing strong positive immunostaining (+++) in the wound healing area (×100). (**C**) Section in the Betadine™-treated group showing mild positive TNF-α immunostaining (+) in the wound healing area (×100). (**D**) Section in the AgNPs-treated group showing negative TNF-α immunostaining (−) in the wound healing area (×100).

Light microscopy of the TNF-α-immunostained skin sections is shown in Figure 15. The AgNPs-treated group showed negative TNF-α immunostaining.

#### 3.6.4. ELISA

The pro-inflammatory cytokines IL-6 and IL-1β significantly decreased (*p* < 0.05) in the Betadine™- and AgNPs-treated groups relative to the control (Table 3). 

#### 3.6.5. qRT-PCR

**Figure 16:**
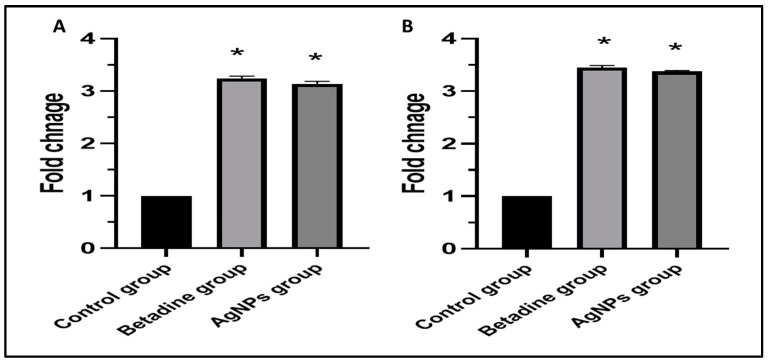
Bar charts represent the fold change in the gene expression of (**A**) fibronectin and (**B**) PDGF genes. The symbol (*) denotes a significant increase (*p* < 0.05) relative to the control group.

Both groups treated with Betadine™ and AgNPs demonstrated a pronounced increase (*p* < 0.05) in fibronectin and PDGF gene expression compared to the control group, as shown in Figure 16.

## 4. Discussion

*C. albicans* pathogenicity is primarily attributed to numerous virulence factors and their escalating resistance to the current antifungals [30]. Thus, the discovery of novel antifungals with activity against both planktonic and biofilm-forming cells is needed to overcome the problematic infections caused by this pathogen. Herein, we synthesized AgNPs from ELLE and investigated their antifungal activity against *C. albicans*.

Using 21 reference chemicals, LC-ESI/MS assessed and quantified the flavonoids and phenolic acids in ELLE. The bioactive chemicals in distinct Gymnosperm species have been documented in a few publications [6,31,32]. The LC-MS/MS analysis detected phenolic compounds claimed to have antimicrobial, antioxidant, and anticancer properties via several pathways [33]. 

Based on our observations of the strong antioxidant potential of ELLE, it not only scavenges free radicals but also breaks them down and reduces ferric ions. *E. laurentianus* have phenolic and flavonoid compounds that possess the ability to form and stabilize AgNPs. These may also be responsible for ELLE-dependent properties. 

AgNPs demonstrated antifungal activity via an agar diffusion assay against *C. albicans* isolates and broth microdilution assay with a range of MICs of 8–256 µg/mL. However, other researchers have assessed the antifungal activity of AgNPs. Xia et al. [34] reported that AgNPs have antifungal activity against *Trichosporon asahii.* Jalal et al. [35] noted the antifungal potential of AgNPs against *C. albicans*, *C. krusei*, *C. tropicalis*, and *C. parapsilosis*. Herein, the AgNPs produced using ELLE have been shown, for the first time, to have antifungal activity both *in vitro* and *in vivo*. Time-kill curves provide a relationship between kill rate and MIC values [36,37]. Increased concentrations of AgNPs decrease the time required for killing fungi. 

The formation of biofilm is an important virulence factor of *C. albicans* which helps in its colonization in human tissues. In addition, it provides the fungal cells with a protective environment in tissues that increases its survival rate compared with planktonic cells [36,37]. The AgNPs reduced the percentage of *C. albicans* isolates, which are capable of forming biofilm strongly and moderately, from 69.23% to 30.77%. SEM analysis showed that the morphology of some *C. albicans* isolates was altered due to treatment with AgNPs. A limited number of studies reported the antibiofilm potential of AgNPs on the *C. albicans* biofilms. Some attempt has been made to explore the mechanism of the antibiofilm action. Różalska et al. [38] proposed that the antibiofilm action was due to the ability of AgNPs to bind and penetrate the biofilm structure, which leads to disrupting the cell membranes. The same study also suggested that AgNPs inhibit biofilm formation by disrupting yeast morphogenesis [38,39]. Several studies have assessed the antibiofilm activity of plant extracts. One reported that biofilm formation was inhibited in *Candida* spp clinical isolates by 65% [8]. 

We tried to reveal the possible mechanisms of the antibiofilm action of AgNPs. The use of qRT-PCR to study the impact on the expression of the genes encoding biofilm formation showed that AgNPs exposure led to the downregulation of the biofilm genes to different extents in the tested isolates. Many genes in *C. albicans* are involved in biofilm development. Some of these genes encode transcription factors that function indirectly to control biofilm formation, such as *BCR1* [40,41]. Others are related to adhesion, which is the first step in biofilm formation, such as surface adhesins *ALS1* [42]. Furthermore, the genes encoding phospholipases, such as *PLB2*, and aspartyl proteases, such as *SAP5*, have an important role in fungal colonization [43]. The mode of action of AgNPs against *C. albicans* isolates was elucidated in previous research. It was reported that AgNPs affect the membrane of the fungal cells and possibly the integrity of the membrane due to increasing the cell permeability and outflow of the cellular proteins, DNA, and RNA [44]. Other researchers reported that the antibiofilm activity is mainly attributed to the inhibition of the adherence of the planktonic cells of fungi to different surfaces [45]. Future studies are needed to elucidate the exact mode of action of AgNPs. 

Animal models can allow efficient assessment of the progression of fungal pathogenesis and host immune responses. Moreover, they can be used to investigate the antifungal properties of certain drug candidates [46]. AgNPs exhibited a promising wound healing potential for the infected rat wounds, as they induced complete epithelization and decreased the TNF-α immunostaining. TNF-α usually increases in non-healing wounds due to oxidative stress and inflammation [47]. Furthermore, the synthesis of collagen fibers was significantly higher in the case of treatment with AgNPs. This is an important finding as the wound healing can be improved via deposition of new collagen into the wound tissues [48]. In addition, it significantly reduced (*p* < 0.05) the levels of the pro-inflammatory cytokines, which cause inflammation and retardation of the wound healing process [49]. In addition, there was a considerable upregulation (*p* < 0.05) of the fibronectin and PDGF genes. Fibronectin and PDGF are essential in improving wound repair and inducing tissue regeneration [50].

## 5. Conclusions

ELLE has a significant content of phenols and flavonoids including compounds such as 3,4-dihydroxybenzoic acid, chlorogenic acid, naringenin, and luteolin. These bioactive constituents are likely to be responsible for forming and stabilizing AgNPs. The antifungal potential of AgNPs green synthesized from ELLE was elucidated. They exhibited in vitro antifungal activity with MIC values of 8 to 256 µg/mL for *C. albicans* clinical isolates.

Significant antibiofilm activity was revealed using crystal violet and SEM. Furthermore, AgNPs substantially down-regulated mRNAs important for biofilm formation in some clinical isolates. Furthermore, AgNPs exhibited a wound healing potential as they induced a complete epithelization, significantly decreased IL-6 and IL-1β levels, and markedly upregulated the genes encoding fibronectin and PDGF. This study opens opportunities to explore the value of AgNPs in the treatment of infections caused by pathogenic fungi like *C. albicans*. Further experimental and clinical studies can now be performed to elucidate the potential of AgNPs as a practical antifungal agent.

## Figures and Tables

**Table 1:** Chemical composition analysis of ELLE’s compounds.

Number	Retention Time (RT)	Compound	Conc. (μg/g)
1	3.85	Gallic acid	61.24
2	5.74	3.4-Dihydroxybenzoic acid	767.72
3	7.26	Catechin	ND
4	7.35	Chlorogenic acid	106.25
5	7.46	Methyl gallate	4.18
6	8.04	Caffeic acid	63.53
7	8.4	Syringic acid	17.10
8	9.52	Coumaric acid	37.46
9	9.56	Vanillin	59.76
10	9.69	Rutin	81.99
11	9.89	Ellagic acid	8.59
12	10.23	Ferulic acid	108.53
13	11.79	Myricetin	ND
14	12.9	Daidzein	ND
15	13.49	Luteolin	486.28
16	13.56	Quercetin	6.94
17	14.18	Cinnamic acid	ND
18	14.95	Naringenin	66,647.08
19	15.01	Apigenin	356.88
20	15.32	Kaempferol	4.05
21	15.6	Hesperetin	14.37

**Table 2:** Impact of AgNPs on biofilm formation by *C. albicans* isolates.

Biofilm Formation	No. of Isolates	
	Before Treatment	After Treatment
No formation	1	3
Weak	3	6
Moderate	5	3
Strong	4	1

**Table 3:** The levels of IL-6 and IL-1β of the wound tissues of the different groups.

Parameters	Control Group	Betadine Group	AgNPs Group
IL-6 (pg/mg tissues)	395.6 ± 4.2	72.8 ± 4.2	70.5 ± 5.5
IL-1β (pg/mg tissues)	471.3 ± 5.4	132.7 ± 6.7	148.8 ± 5.4

## Data Availability

Data are contained within the article and Supplementary Materials.

## Supplementary Materials

The following supporting information can be downloaded at https://www.mdpi.com/article/10.3390/jof8101005/s1: Table S1. Categories of biofilm-forming *C. albicans* isolates. Table S2. Sequences of primer used in qRT-PCR in vitro. Table S3. Sequences of primers utilized in qRT-PCR in vivo. Table S4. MIC values. Figure S1. The wound healing process of the different groups on days 0, 3, and 7.
