# Supplementary material for: Silver Nanoparticles Prepared Using Encephalartos laurentianus De Wild Leaf Extract Have Inhibitory Activity against Candida albicans Clinical Isolates
Source: J Fungi (Basel). 2022 Sep 25;8(10):1005. doi: 10.3390/jof8101005 (PMC9604723; doi:10.3390/jof8101005)
Supplement: Supplementary file 1 [file jof-08-01005-s001.zip › jof-1883593-supplementary.pdf]

Table S1. Categories of biofilm forming *C. albicans* isolates.

Biofilm forming ability	OD values*
**Non-biofilm forming**	OD ≤ ODc
**Weak biofilm forming**	OD> ODc ≤ 2× ODc
**Moderate biofilm-forming**	OD> 2× ODc ≤ 4× ODc
**Strong biofilm-forming**	OD > 4× ODc

*cut-off OD (ODc) is the mean OD of the negative control (SDB only without fungi) plus three times standard deviations (SD).

Table S2. Sequences of primer used in qRT-PCR *in vitro*.

Gene name	Sequence 5′- 3′
ACT1 **(reference gene)**	F- TTTCATCTTCTGTATCAGAGGAACTTAT<br>R- ATGGGATGAATCATCAAACAAGAG
BCR1	F- AATGCCTGCAGGTTATTTGG<br>R- TTTTAGGTGGTGGTGGCAAT
PLB2	F- TGAACCTTTGGGCGACAACT<br>R- GCCGCGCTCGTTGTTAA
ALS1	F- CAACAGGCACCTCAGCATCTAC<br>R- CTCCACCAGTAACAGATCCACTAGTAA
SAP5	F- CCAGCATCTTCCCGCACTT<br>R- GCGTAAGAACCGTCACCATATTTAA

Table S3. Sequences of primers utilized in qRT-PCR *in vivo*.

Primer	Sequence
**GAPDH**	F 5'-CAGCAATGCATCCTGCAC-3'<br>R 5'-GAGTTGCTGTTGAAGTCACAGG-3'
**Fibronectin**	F 5′-GAGCTATCCATTTCACCTTCAGA-3'<br>R 5′-TTGTTCGTAGACACTGGAGAC-3'
**PDGF**	F 5'-TGCCAGAGCCTGCTCTTAAC-3'<br>R 5'-GATGCCACGGAGATAAGCGA-3'

Table S4. Values of MICs of ELLE against the tested *C. albicans* clinical isolates.

Isolate code	MIC (µg/mL)	Isolate code	MIC (µg/mL)
**C1**	8	C8	16
**C2**	16	C9	16
**C3**	8	C10	8
**C4**	64	C11	32
**C5**	32	C12	128
**C6**	8	C13	256
**C7**	256		

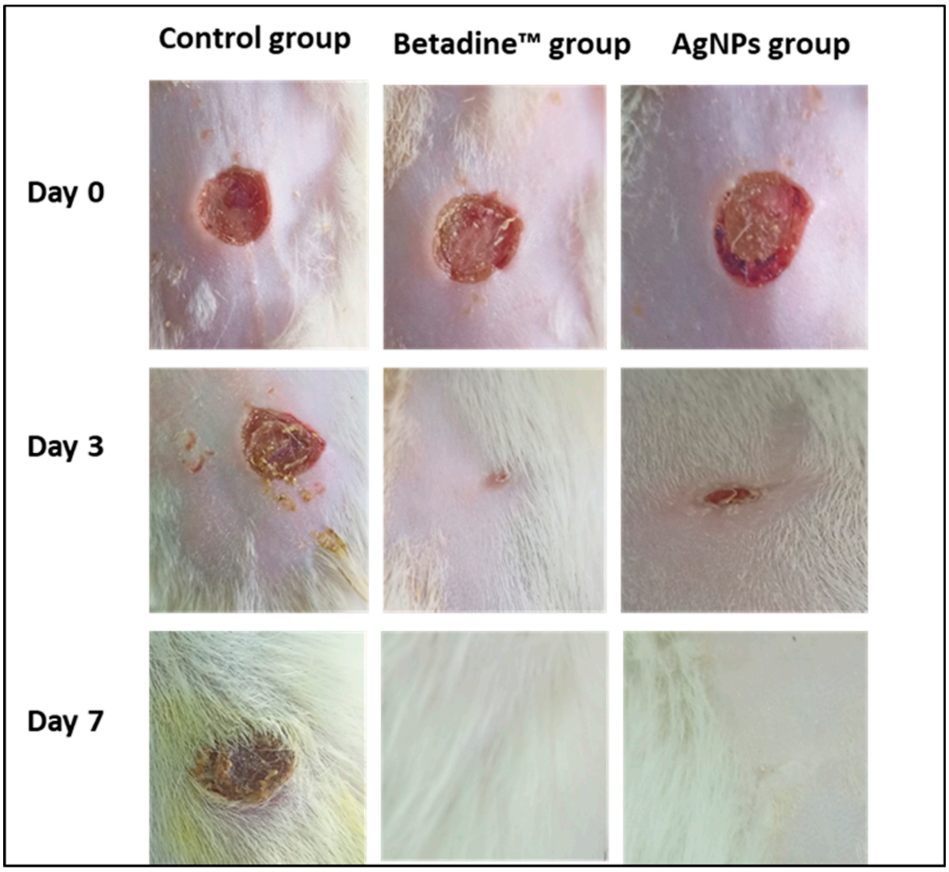

Figure S1. The wound healing process of the different groups on days 0, 3, and 7.
